# Circulating MicroRNAs Characterizing Patients with Insufficient Coronary Collateral Artery Function

**DOI:** 10.1371/journal.pone.0137035

**Published:** 2015-09-02

**Authors:** Nazanin Hakimzadeh, A. Yaël Nossent, Anja M. van der Laan, Stephan H. Schirmer, Maurice W. J. de Ronde, Sara-Joan Pinto-Sietsma, Niels van Royen, Paul H. A. Quax, Imo E. Hoefer, Jan J. Piek

**Affiliations:** 1 Department of Biomedical Engineering & Physics, Academic Medical Center, University of Amsterdam, Amsterdam, The Netherlands; 2 Department of Surgery, University Medical Center, Leiden, The Netherlands; 3 Einthoven Laboratory for Experimental Vascular Medicine, Leiden, University Medical Center, Leiden, The Netherlands; 4 Department of Cardiology, Academic Medical Center, University of Amsterdam, Amsterdam, The Netherlands; 5 Department of Cardiology, Klinik für Innere Medizin III, Universität des Saarlandes, Homburg/Saar, Germany; 6 Department of Vascular Medicine, Academic Medical Center, University of Amsterdam, Amsterdam, The Netherlands; 7 Department of Cardiology, VU University Medical Center, Amsterdam, The Netherlands; 8 Laboratory of Experimental Cardiology, University Medical Center Utrecht, Utrecht, The Netherlands; National University of Singapore, SINGAPORE

## Abstract

**Background:**

Coronary collateral arteries function as natural bypasses in the event of coronary obstruction. The degree of collateral network development significantly impacts the outcome of patients after an acute myocardial infarction (AMI). MicroRNAs (miRNAs, miRs) have arisen as biomarkers to identify heterogeneous patients, as well as new therapeutic targets in cardiovascular disease. We sought to identify miRNAs that are differentially expressed in chronic total occlusion (CTO) patients with well or poorly developed collateral arteries.

**Methods and Results:**

Forty-one CTO patients undergoing coronary angiography and invasive assessment of their coronary collateralization were dichotomized based on their collateral flow index (CFI). After miRNA profiling was conducted on aortic plasma, four miRNAs were selected for validation by real-time quantitative reverse transcription polymerase chain reaction in patients with low (CFI<0.39) and high (CFI>0.39) collateral artery capacity. We confirmed significantly elevated levels of miR423-5p (p<0.05), miR10b (p<0.05), miR30d (p<0.05) and miR126 (p<0.001) in patients with insufficient collateral network development. We further demonstrated that each of these miRNAs could serve as circulating biomarkers to discriminate patients with low collateral capacity (p<0.01 for each miRNA). We also determined significantly greater expression of miR30d (p<0.05) and miR126 (p<0.001) in CTO patients relative to healthy controls.

**Conclusion:**

The present study identifies differentially expressed miRNAs in patients with high versus low coronary collateral capacity. We have shown that these miRNAs can function as circulating biomarkers to discriminate between patients with insufficient or sufficient collateralization. This is the first study to identify miRNAs linked to coronary collateral vessel function in humans.

## Introduction

Collateral artery growth, a process known as *arteriogenesis* [1], provides an alternative route for blood perfusion in the event of obstructive coronary artery disease. Coronary artery disease (CAD) patients with a well-developed collateral network exhibit better preservation of myocardial function and are less vulnerable to adverse cardiac events, with reduced mortality [2–4].

Previous studies have shown differences in gene expression at messenger RNA (mRNA) level between CAD patients with poor versus well-developed coronary collateral arteries [5, 6]. However, there is currently limited information on microRNA (miRNA) expression in CAD patients with varying degree of collateral artery formation.

In recent years, miRNAs have been identified as new targets for pharmaceutical intervention. MiRNAs are small non-coding RNAs (~22 nucleotides in length) that suppress translation or induce degradation of downstream mRNA targets, thereby modulating gene expression at a post-transcriptional level [7]. Currently, there is limited knowledge about miRNAs that play a role in arteriogenesis and vascular remodeling [8, 9]. Recent studies have identified miRNAs as suitable biomarkers to discriminate patients with cardiovascular diseases, including heart failure, stable CAD, as well as acute myocardial infarction (AMI) [10]. In this study, we sought to identify circulating miRNAs that are differentially expressed in chronic total occlusion (CTO) patients with poor and well-developed coronary collateral arteries. In addition, we aimed to determine which miRNAs would be suitable biomarkers to discriminate CTO patients with either high or low collateral artery capacity.

## Methods

### Patient Population

This study was conducted in accordance with the Declaration of Helsinki. The institutional medical ethics committee of the Academic Medical Center of the University of Amsterdam approved the study protocol, and all patients gave written informed consent. The patient study population and protocol have been described previously [6, 11]. Briefly, 41 Caucasian patients that underwent successful elective percutaneous coronary intervention (PCI) of a CTO were included. Patients were deemed an eligible candidate for the study if they had symptoms of angina pectoris for ≥4 weeks and a CTO of a coronary artery. Exclusion criteria included previous myocardial infarction, cardiac surgery, depressed left ventricular function, diabetes mellitus and inflammatory or neoplastic disease. Due to the potential influence of diabetes mellitus on collateral vessel formation [12] and miRNA expression patterns [13], patients with diabetes mellitus were excluded such that the expression level of miRNAs associated with collateral vessel capacity could be examined independently. Laboratory values collected for each patient included leukocyte counts (10^9^/L) as well as leukocyte subset counts (thrombocytes, neutrophils, eosinophils, basophils, lymphocytes, monocytes). Collateral flow index (CFI) was measured as described previously [6, 11].

### Plasma collection and storage

Aortic blood (7mL) was withdrawn from the aortic root from all patients at the beginning of the cardiac catheterization protocol. Blood was transferred into sterile citrate tubes (Vacutainer^TM^, Beckton Dickinson), placed on ice and centrifuged (1500g, 20 minutes). Plasma was stored in RNase free vials at -80°C until usage.

### Real Time-PCR multiplex

Expression profiling of nearly 750 different miRNAs was conducted by a real time polymerase chain reaction (RT-PCR) multiplex assay. For this plasma samples from 3 patients with either high CFI (CFI>0.37) or low CFI (CFI<0.31) were pooled together into one sample. Six pooled samples from patients with high collateral capacity, and 6 pooled samples from those with low collateral capacity were included.

RT-PCR multiplex analysis was conducted by Exiqon Services, Denmark. Total RNA was extracted from plasma using the Qiagen miRNeasy Mini Kit, according to the manufacturer’s protocol. Isolated RNA was reverse transcribed, complementary DNA (cDNA) synthesized and assayed in PCR reactions using the miRCURY LNA Universal RT miRNA PCR, Polyadenylation and cDNA synthesis kit (Exiqon) based on the manufacturer’s protocols. Each miRNA was assayed once by quantitative PCR (qPCR) on the miRNA Ready-to-Use PCR, Human panel I and panel II. Negative controls excluding template from the reverse transcription reaction were also included. Amplification was performed in a LightCycler 480 RT-PCR System (Roche). Amplification curves were analyzed using the Roche LC software. Amplification efficiency was determined using algorithms similar to the LinReg software. Using NormFinder software the best normalizer was found to be the average of assays detected in all samples. All data was normalized to the average of assays detected in all samples (average–assay Cp).

### Validation of RT-PCR multiplex results

Total RNA was isolated from plasma samples of all 41 patients using the mirVana PARIS kit (Life Technologies) according to the manufacturer’s protocol. Patients were dichotomized into low (CFI<0.39) and high (CFI>0.39) collateral capacity. This value was chosen based on the study of van der Hoeven et al., whereby 0.39 was the mean CFI value in a large CTO patient population (n = 295)[12]. Total RNA was also isolated from plasma acquired by venous puncture from 19 healthy volunteers using TRIzol (Ambion), in line with the manufacturer’s instructions.

MiRNA quantification was performed on all individual samples (in triplicate) using TaqMan microRNA assays (Applied Biosystems), according to manufacturer's protocol. QPCRs were run on a 7900HT Fast RT-PCR System (Applied Biosystems), and amplification efficiencies were checked by standard curves. Based on the data collected from the RT-PCR multiplex, three miRNAs were selected to be validated as stably expressed controls (mir15a, mir16, mir223). Normalization of data was performed with the most stably expressed endogenous control.

### Statistical analyses

Values are expressed as mean ± standard deviation (SD). Statistically significant outliers in miRNA RT-PCR data were identified using a Grubb’s test, and subsequently excluded. As the validation experiments generated values that were derived from individual RT-PCR reactions and each of the miRNAs are transcribed from different genes and therefore they are independently regulated, expression level of each respective miRNA was treated as an independent variable. As a result, in the event that an outlier was identified for one miRNA, the data for this patient was not excluded for all other miRNAs examined. MiRNA measurements in a few patient samples were omitted for select miRNAs as there were undetectable plasma levels of the respective miRNAs. Student’s unpaired two-tailed t-test analysis was used to compare two groups with a normal distribution, and the Mann-Whitney U-test was used for data with a non-normal distribution. Where applicable, Welch’s correction was used to consider unequal variances. Fisher’s exact test was used to determine statistical differences in categorical groups. Correlations were calculated with a Pearson correlation for normally distributed data, and the Spearman correlation was used for non-normally distributed data. Receiver operator characteristic (ROC) curves were analyzed to assess sensitivity and specificity of each miRNA. This statistical test depicts the sensitivity and specificity of a variable (individual miRNA) to discriminate between one of two outcomes, these outcomes being either high or low collateral capacity. To consider multiple parameters, a multivariate logistic regression was performed with each individual miRNA together with age and gender. The predicted probabilities from the multivariate logistic regression model were used to generate ROC curves. Where applicable, the optimal diagnostic point to discriminate patients with a CFI>0.39 was determined at a cut-off value with the largest Youden’s index (Sensitivity+Specificity–1). A likelihood ratio (LR) was also used to examine the clinical impact to assess the likelihood that a patient with the respective cut-off value has low (CFI<0.39; LR-) or high (CFI>0.39; LR+) collateral capacity. Statistical analysis was conducted using GraphPad Prism 5 and IBM SPSS Statistics 20, whereby a p-value <0.05 was considered statistically significant.

## Results

### Patient characteristics

**Fig 1:**
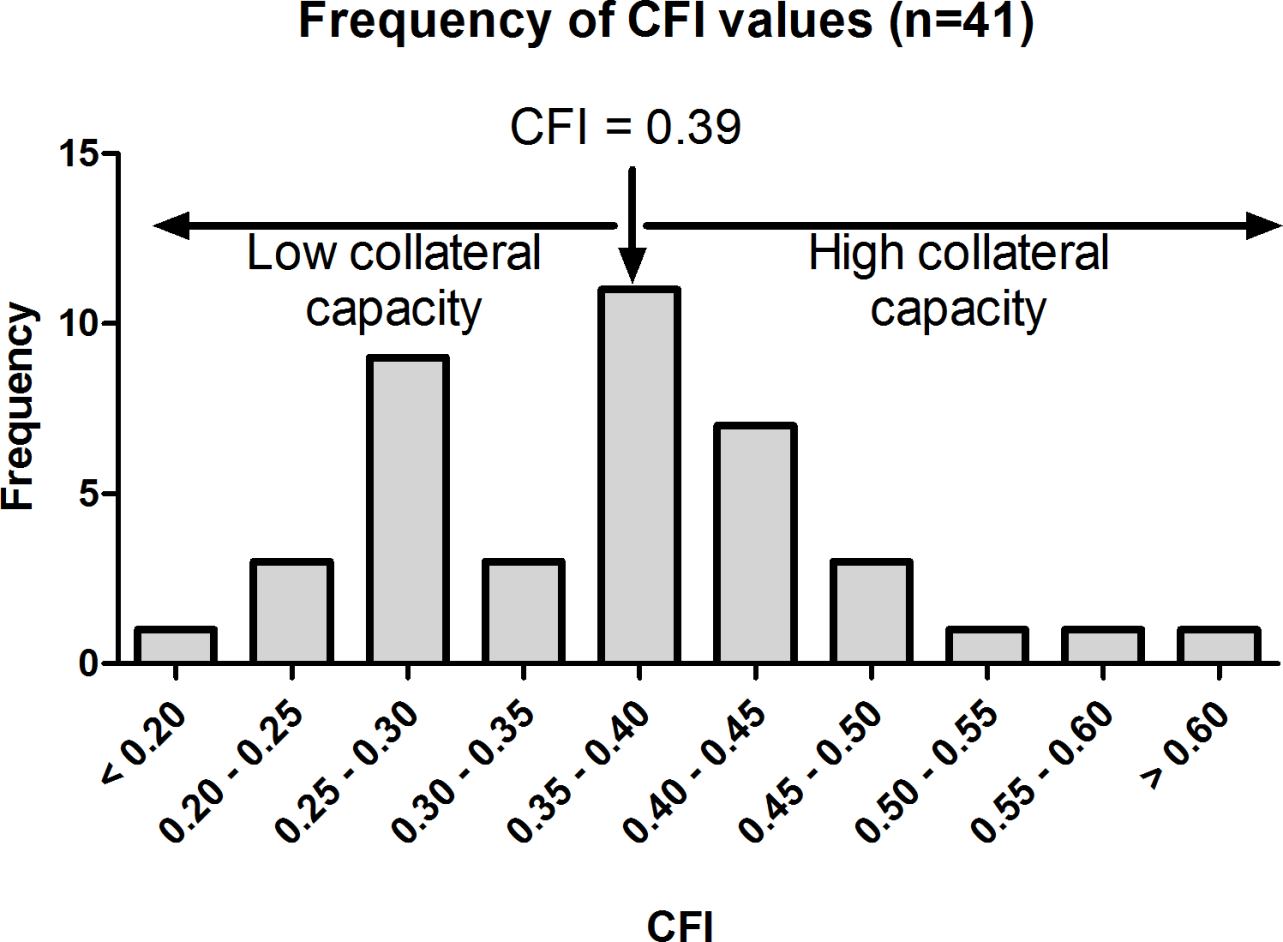
Frequency distribution of collateral flow index (CFI) in patient group (n = 41). Patients were dichotomized into two groups, low (CFI <0.39) and high (CFI>0.39) collateral capacity

The mean age of the patients included in this study was 59±11 years and 30 (71%) were male (see Table 1 for patient characteristics). All patients underwent invasive CFI measurements, whereby a mean CFI of 0.36±0.016 was calculated. The frequency distribution of CFI values is displayed in Fig 1.

**Table 1:** Patient characteristics.

Characteristic	CFI <0.39 (n = 27)	CFI >0.39(n = 14)	p-value
CFI, mean ± SD	0.31 ± 0.059	0.46 ± 0.080	< 0.0001
Age (years), mean ± SD	58 ± 12	60 ± 9	0.58
Male gender, n (%)	22 (81)	8 (57)	0.14
BMI (kg/m^2^), mean ± SD	26.9 ± 3.33	27.8 ± 2.58	0.35
***Coronary Risk Factors***			
Hypertension, n (%)	14 (52)	10 (71)	0.32
Family history of CAD, n (%)	14 (52)	8 (57)	1.00
Hypercholesterolemia, n (%)	8 (30)	9 (64)	<0.05
Current smoker, n (%)	4 (15)	1 (7.1)	0.64
Past smoker, n (%)	16 (59)	7 (50)	0.74
***Target vessel***			
LAD, n (%)	7 (26)	5 (36)	0.72
RCA, n (%)	18 (66)	6 (43)	0.19
RCX, n (%)	2 (7.4)	3 (21)	0.32
***Duration of anginal symptoms***			
< 3 months, n (%)	7 (26)	3 (21)	1.00
3 months to 1 year, n (%)	11 (41)	7 (50)	0.74
> 1 year, n (%)	8 (30)	3 (21)	0.72
***Medication***			
Aspirin, n (%)	24 (89)	13 (93)	1.00
ACE-inhibitors/ARBs, n (%)	10 (37)	7 (50)	0.51
β - blockers, n (%)	22 (81)	12 (86)	1.00
Statins, n (%)	25 (93)	13 (93)	0.60
Clopidogrel, n (%)	19 (70)	8 (57)	0.49
Calcium Antagonists, n (%)	5 (18.5)	4 (29)	0.69
Nitrates, n (%)	15 (56)	7 (50)	0.75
***Leukocytes***			
WBC (10^9^/L ± SD)	7.0 ± 1.8	6.9 ± 1.3	0.9
Thrombocytes (10^9^/L ± SD)	220 ± 53	249 ± 71	0.1
Neutrophils (10^9^/L ± SD)	4.4 ± 1.4	4.5 ± 0.95	0.8
Eosinophils (10^9^/L ± SD)	0.17 ± 0.10	0.17 ± 0.14	0.8
Basophils (10^9^/L ± SD)	0.035 ± 0.031	0.031 ± 0.012	0.7
Lymphocytes (10^9^/L ± SD)	1.9 ± 0.57	1.7 ± 0.61	0.4
Monocytes (10^6^/L ± SD)	525 ± 147	462 ± 148	0.2

Patient characteristics were comparable in patients with low (CFI<0.39) and high (CFI>0.39) collateral capacity, with the exception of a greater incidence of hypercholesterolemia in the high collateral capacity group. ACE, Angiotensin converting enzyme; ARBs, angiotensin receptor blockers; BMI, body mass index; CAD, coronary artery disease; CFI, collateral flow index; LAD, left anterior descending; RCA, right coronary artery; RCX, right circumflex.

### MiRNA RT-PCR Multiplex Results

**Fig 2:**
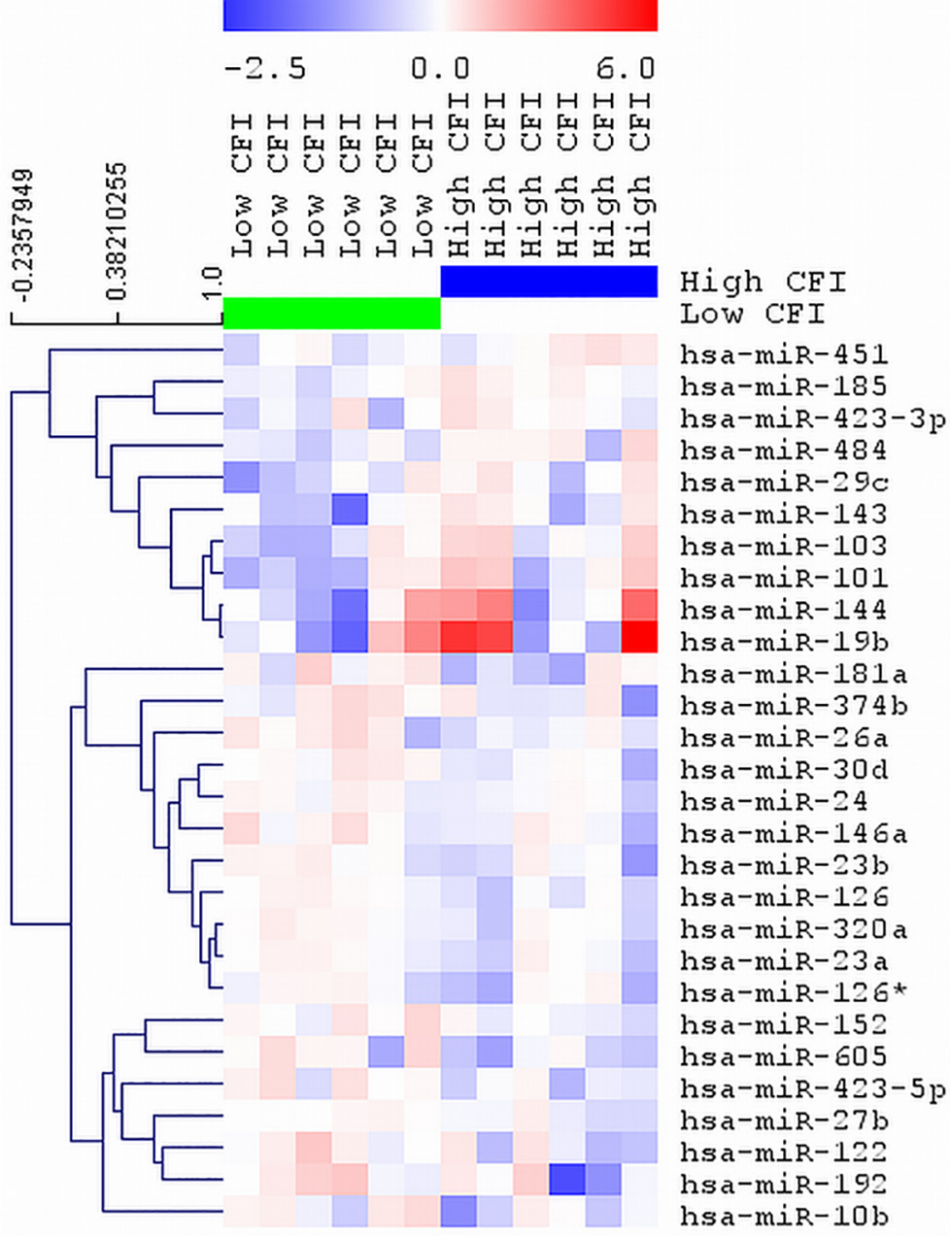
Differential microRNA expression in patients with high versus low CFI. Heat map and supervised hierarchical clustering of the top 28 microRNAs with the lowest p-value across all samples. Each row represents one microRNA and each column represents one sample. Each sample consists of pooled plasma samples from 3 patients with either high CFI or low CFI, resulting in a total of 12 samples. The color scale shows the relative expression level of a microRNA across samples, where red color depicts an expression level above mean and blue color represents down regulated expression. CFI: collateral flow index.

The expression profiles of ~750 miRNAs were assessed in plasma from patients with high and low collateral capacity. Fig 2 displays a heat map showing the top 28 miRNAs with highest differential expression. Significantly greater miR126, miR30d, miR423-5p and miR10b expression (p-value of 0.011, 0.029, 0.050 and 0.051, respectively) were seen in the plasma of patients with low collateral capacity compared to those with high collateral capacity. We sought to validate the expression pattern of these select miRNAs by qPCR.

### Validation of differential microRNA expression

Based on the miRNA profiling data from the RT-PCR multiplex, three miRNAs were selected as potential reference miRNA candidates because of their stable expression in patients with low and high collateral capacity (miR15a, miR16 and miR223). These were selected based on their low coefficient of variation (Cv) and non-significant difference in expression levels between both patient groups. We verified stable expression levels of miR15a, miR16 and miR223 by qPCR based on their cycle threshold (Ct) values and determined the lowest Cv in miR223 (S1 Fig). Thus, miR223 was used as a reference miRNA.

**Fig 3:**
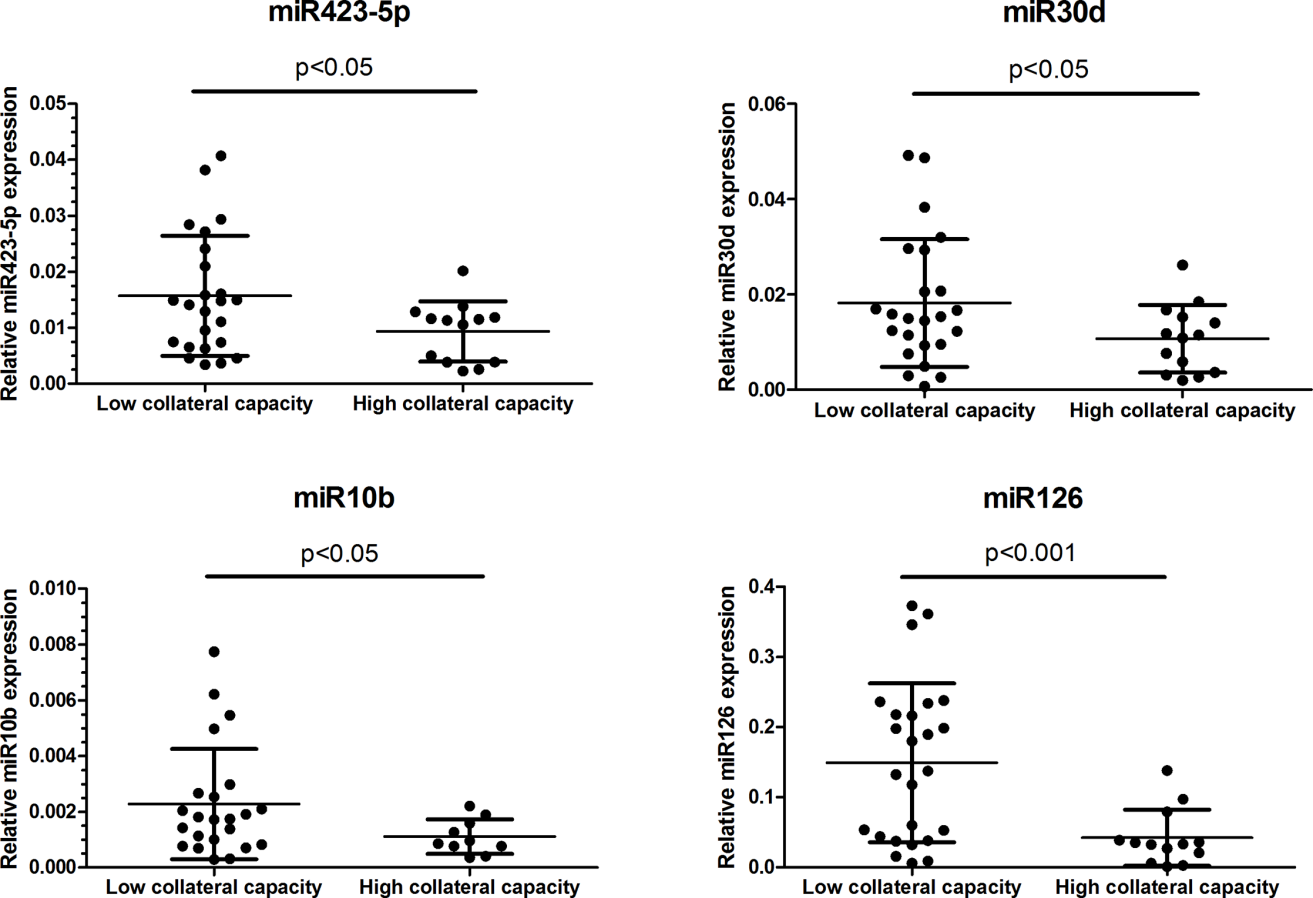
Elevated expression of select microRNAs in patients with low collateral capacity. Values are based on qPCR measurements. Data are presented as mean ± SD. CFI: collateral flow index.

Using miR223 for normalization of miRNA expression levels, we confirmed significantly greater miR423-5p (p<0.05), miR30d (p<0.05), miR10b (p<0.05) and miR126 (p<0.001) expression in patients with low collateral capacity (Fig 3). There was no significant correlation between the expression levels of these miRNAs (miR423-5p, miR10b, miR30d or miR126) with CFI (S2 Fig).

### MicroRNAs for discrimination of patients with high or low collateral capacity

**Fig 4:**
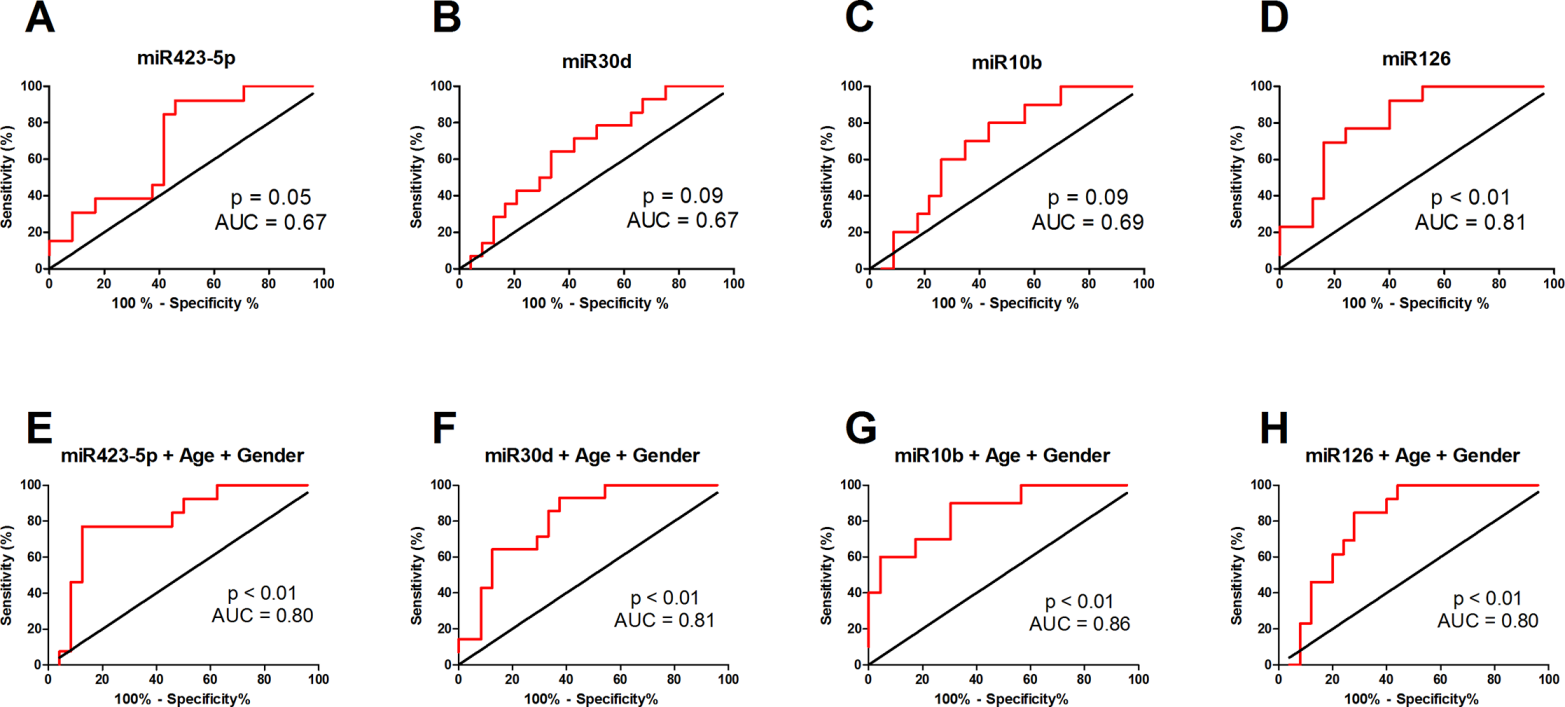
Diagnostic potential of miRNAs. Receiver operator characteristic curve analysis of individual miRNAs (A: miR423-5p; B: miR30d; C: miR10b; D: miR126) and multivariate logistic regression models of individual microRNAs together with age and gender (E: miR423-5p; F: miR30d; G: miR10b; H: miR126) to discriminate between high or low collateral capacity patients. Red line depicts sensitivity (%) as a function of 1- specificity (%). The black line depicts the identity line. The greater the area between the ROC curve (red) and identity line (black), the more accurate the test and the larger the discriminatory power of the test. Multivariate logistic regression models with age and gender increase the area under the curve (AUC) of each miRNA, and thus improve their power to discriminate between patients with either high or low collateral capacity.

To determine if these selected miRNAs could be used to discriminate between patients with high or low collateral capacity, we conducted ROC curve analysis. MiR126 was a significant predictor of collateral capacity with an AUC of 0.81 (Fig 4A–4D, Table 2). The cut-off value of miR126 to discriminate between patients with high or low collateral capacity was determined to be <0.037, with a LR+ of 4.3. Nonetheless, recent studies have shown that age and gender can also influence miRNA expression levels and collateral artery formation [14–17]. Thus, using a multivariate logistic regression model with age and gender, we found that all miRNAs showed AUC greater than 0.8 (Fig 4E–4H, Table 2).

**Table 2:** Receiver operating characteristic curves.

miRNA	AUC	95% CI	*P*-value	Cut-off	Sensitivity (%)	95% CI	Specificity (%)	95% CI	LR+	LR-
miR423-5p	0.67	0.52 to 0.87	0.05	N/A	N/A	N/A	N/A	N/A	N/A	N/A
miR30d	0.67	0.49 to 0.84	0.09	N/A	N/A	N/A	N/A	N/A	N/A	N/A
miR10b	0.69	0.50 to 0.87	0.09	N/A	N/A	N/A	N/A	N/A	N/A	N/A
miR126	0.81	0.68 to 0.95	<0.01	<0.037	69	39–91	84	64–95	4.3	2.7
miR423-5p + Age + Gender*	0.80	0.65 to 0.95	<0.01	<0.57	77	46–95	87	68–97	6.1	3.8
miR30d + Age + Gender*	0.81	0.68 to 0.95	<0.01	<0.52	64	35–87	87	68–97	5.1	2.4
miR10b + Age + Gender*	0.86	0.72 to 0.99	<0.01	<0.77	90	55–100	70	47–87	3.0	7.0
miR126 + Age + Gender*	0.80	0.66 to 0.94	<0.01	<0.56	85	55–98	72	51–88	3.0	4.7

Properties of receiver operator characteristic curves shows that miR126 levels can significantly discriminate between patients with low CFI (<0.39) versus high CFI (>0.39), with a p-value <0.01. In addition, in a multivariate logistic regression model with age and gender, each of the select miRNAs show significant predictive power to discriminate between patients with high or low collateral capacity.

*Multivariate logistic regression model. AUC, area under curve; CI, confidence interval; CFI: collateral flow index; LR, likelihood ratio; miRNA, microRNA; N/A, not applicable.

### Association of microRNAs and circulating leukocyte subsets

Circulating leukocytes play an important role in collateral vessel formation [18]. Previous studies have shown that good collateral development is associated with low levels of circulating leukocytes [12], and higher levels of circulating monocytes in CAD patients [19]. Thus, we examined whether there were correlations between the levels of each respective miRNA to circulating leukocyte subsets. We found significant correlations between the level of miR10b and lymphocytes (p<0.05), as well as between the level of miR10b and monocytes (p<0.05) (S1 Table).

### Basal expression of microRNAs in healthy individuals relative to CTO patients

**Fig 5:**
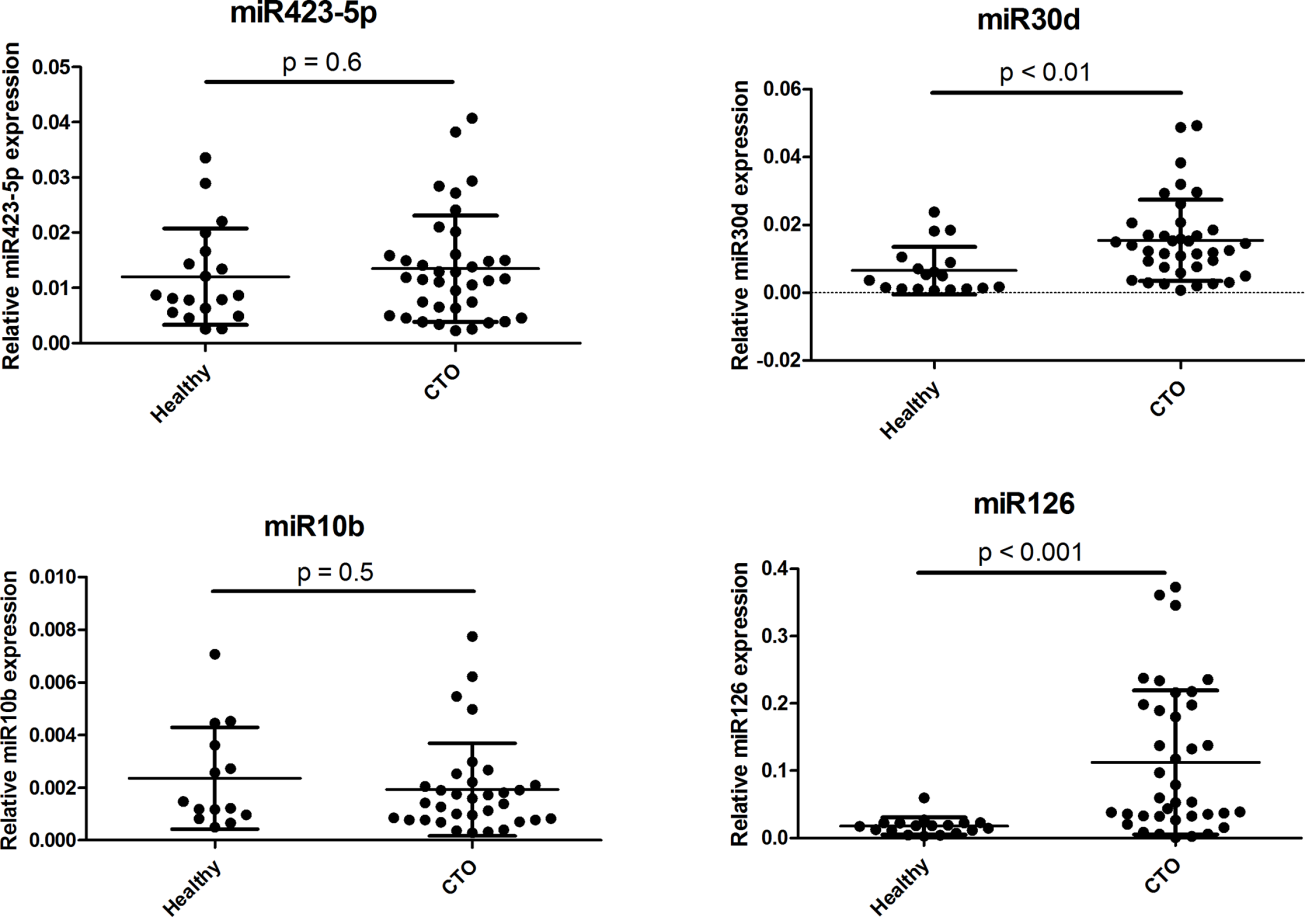
Expression of microRNAs in healthy individuals and CTO patients. Values are based on qPCR measurements. Data are presented as mean ± SD. CTO: chronic total occlusion.

We also sought to compare the expression level of these miRNAs (miR423-5p, miR30d, miR10b and miR126) between healthy individuals (n = 19) relative to our CTO patient population (n = 41). The mean age of our healthy population was 51±4, and all participants were male. We compared the expression of the select miRNAs in healthy individuals to all CTO patients without dichotomization based on their CFI value, due to lack of CFI measurements in healthy volunteers. Interestingly, miR30d and miR126 showed significantly greater expression in CTO patients relative to healthy individuals (p<0.01 and p<0.001, respectively; Fig 5), wherebymiR126 showed over two-fold greater expression in CTO patients.

## Discussion

The present study shows that miR423-5p, miR30d, miR10b and miR126 are up-regulated in plasma of CTO patients with low coronary collateral artery capacity. We have also determined that these miRNAs can be used as circulating biomarkers to discriminate between patients with insufficient collateral arteries relative to patients with sufficient collateral artery capacity. Compared to healthy individuals, miR30d and miR126 show elevated expression in CTO patients.

Previous studies have shown that patients with sufficient versus insufficient coronary collateral artery development display distinctively different gene expression profiles at mRNA level. Transcriptional profiling of peripheral blood monocytes revealed 244 differentially expressed genes in patients with poor versus well-developed collateral networks [5, 6, 11]. Patients with low collateral capacity displayed heightened activation of inhibitory pathways, based on increased interferon-β [5] and galectin-2 [11] mRNA expression in peripheral blood monocytes. In addition, it was shown that patients with diminished collateral capacity displayed a distinct genetic polymorphism associated with galectin-2 mRNA expression in peripheral blood monocytes [11].

Based on these previous findings, we hypothesized that patients with varying degrees of collateral artery capacity also display distinct miRNA expression profiles. Interestingly, there was no linear correlation between the expression levels of these miRNAs with CFI. This is supportive of previous findings demonstrating distinct gene expression profiles in CAD and CTO patients dichotomized based on their CFI value [5, 6, 11], suggesting the presence of two distinct patient groups. The miRNAs we have identified to be linked with low collateral artery capacity have never been previously linked with collateral vessel function. It is unclear if these miRNAs play a direct role in collateral vessel growth, or perhaps are up-regulated as a result of other active pathways that in turn impede collateral vessel growth. MiR423-5p and miR126 have been previously linked to heart failure [17] and atherosclerosis progression [20]. MiR423 is part of the let-7 family of miRNAs which have been suggested to suppress expression of their downstream target hepatic nuclear factor 4 alpha (HFN4A), and thereby promote stem cell self-renewal [21]. MiR126 has also been shown to prevent atherosclerosis formation by promoting endothelial cell proliferation and turnover, through the suppression of the Notch1 inhibitor delta-like 1 homolog (Dlk1) [20]. There is limited information directly linking miR10b and miR30d to heart disease. MiR10b has been validated as a circulating biomarker to identify patients with primary and metastatic breast cancer [22]. More recently miR10b has been identified as a novel regulator of brain-derived neurotrophic factor (BDNF) [23]. BDNF is important for angiogenesis in the myocardium [24], and has been associated with cardiovascular risk factors [25]. MiR30d is known to promote cardiomyocyte pyroptosis through the suppression of foxo3a; cardiomyocyte pyroptosis is pro-inflammatory programmed cell death of cardiomyocytes [26].

In this study we have identified miR423-5p, miR30d, miR10b and miR126 as circulating biomarkers to discriminate between patients with low versus high collateral artery capacity. This may be valuable for identifying patients at higher risk of complications in the occurrence of acute coronary events. The beneficial effect of a vast collateral network is known to improve survival of patients suffering from CAD [2–4, 27]. Currently there are no known circulating biomarkers to distinguish between these patient groups. As a result, it is not possible to compare the discriminatory power of these miRNAs to other biomarkers. Clinical parameters associated with collateral vessel function have been recently described [12]. However, identification of miRNAs associated with high or low collateral vessel function in humans has never been previously described.

With the exception of miR126, the other miRNAs described in this study (miR423-5p, miR10b, miR30d), displayed significant discriminatory power only in a multivariate logistic regression model with age and gender. Consistent with other studies, age and gender dependent intrinsic differences in miRNA expression have been shown previously [16, 17, 28]. Similarly patients with older age also show diminished collateral artery capacity relative to their younger counterparts [14, 15].

Circulating leukocytes, particularly monocytes, play a crucial role in modulating collateral vessel growth [18]. Thus, we sought to determine if there was an association between the level of circulating leukocyte subsets and the selected miRNAs. We noted an association between miR10b and circulating monocytes along with lymphocytes. However, no other associations were found between the level of miR423-5p, miR30d or miR126 and the level of circulating leukocyte subsets. This can likely be attributed to the lack of secretion of these miRNAs by circulating leukocytes. MiR30d has shown to be secreted by cardiomyocytes [26], while miR423-5p has been linked to cardiac origin [29]. It is well known that miR126 is abundantly expressed by endothelial cells and is not generated by smooth muscle cells or cardiomyocytes [30]. Platelets have been deemed as a major source of circulating miR126, whereby aspirin treatment has been shown to reduce circulating levels of miR126 [31]. Interestingly, in the current study population 90% of patients were being treated with aspirin and yet patients with poor collateral network displayed elevated levels of miR126 relative to patients with well-developed collateral vessels. This suggests an alternative source for miR126. Previous studies have shown that miR126 is enriched in tissues with a large vascular component, with highest expression in the heart and lung [30].

Although miR126 is perhaps the most extensively studied microRNA in relation to angiogenic vascular growth, this is the first study to link miR126 to the extent of collateral vessel function in humans. MiR126 has been identified as having pro-angiogenic effects and is important for maintaining vascular integrity [30, 32, 33]. Van Solingen et al. investigated the effects of antagomir silencing of miR126 on arteriogenesis and angiogenesis by femoral artery ligation in mice [33]. However, no change in collateral vessel growth was seen in antagomir-126 treated mice relative to controls. Nonetheless, in CTO patients we have noted significantly greater levels of miR126 in patients with insufficient collateralization.

### Clinical Implications

Currently the discrimination between patients with sufficient versus insufficient collateral network relies on invasive intracoronary CFI measurements. Angiography grading can also provide semi-quantitative evaluation of the presence of recruitable collateral vessels [18]. However, angiography is limited to the detection of collateral vessels above 100μm in diameter [34]. In addition, both CFI measurements and angiography require intracoronary catheterization. The use of circulating biomarkers, such as the ones identified in this study, could prove to be immensely valuable in providing a simple and less invasive means of characterizing the coronary collateral capacity of patients. Identification of patients with low collateral capacity is important as these patients are more vulnerable to mortality and have a higher risk of complications in the incident of an acute coronary event.

### Study Limitations

Although we have identified 4 miRNAs associated with coronary collateral vessel capacity, we are limited by a lack of mechanistic insight as to whether these miRNAs are directly involved in collateral vessel growth. We cannot exclude the possibility that these miRNAs are detected in elevated levels in patients with low collateral capacity due to other active pathways that inhibit collateral vessel growth. Nonetheless, this study provokes future work in a larger patient cohort, investigating the source of these miRNAs, as well as examining their exact involvement in collateral vessel growth. Furthermore, as dichotomization of patients into low versus high collateral capacity is entirely dependent on intracoronary measurements of CFI, it is not feasible to examine the level of these miRNAs in healthy control groups with high or low collateral capacity. However, we did note significantly elevated levels of miR30d and mir126 in CTO patients relative to healthy individuals.

## Conclusions

In conclusion, we have identified circulating miRNAs associated with insufficient collateral artery function (miR423-5p, miR10b, miR30d and miR126) in CTO patients. We have also determined that these select miRNAs are suitable circulating biomarkers to discriminate between patients with well versus poorly developed collateral arteries, prompting future studies with a larger patient cohort.

## Supporting Information

S1 Fig MicroRNA candidates for selecting an endogenous control. Cycle threshold values of three reference miRNA candidates (A: miR15a; B: miR16; C: miR223) in patients with low (CFI < 0.39) and high (CFI > 0.39) collateral capacity, as well as healthy individuals. MiR223 demonstrates most stable expression between the two patient groups and healthy controls, with the lowest coefficient of variation (Cv) values. Data are presented as mean ± SD. CFI: collateral flow index; miRNA: microRNA
(TIF)

S2 Fig Correlation between CFI and respective miRNAs in all 41 CTO patients. No significant correlation seen between relative expression levels of selected microRNAs (A: miR423-5p, B: miR30d, C: miR10b, D: miR126) and collateral flow index (CFI) values.
(TIF)

S1 Table Correlation between leukocytes and respective miRNAs in all CTO patients (n = 41). Correlation of circulating miRNAs with circulating leukocyte groups. Significant correlation was noted between miR10b and lymphocyte levels as well as between miR10b and monocytes.
(DOCX)
