# Supplementary figures and images for: Circulating MicroRNAs Characterizing Patients with Insufficient Coronary Collateral Artery Function
Source: PLoS One. 2015 Sep 2;10(9):e0137035. doi: 10.1371/journal.pone.0137035 (PMC4558025; doi:10.1371/journal.pone.0137035)

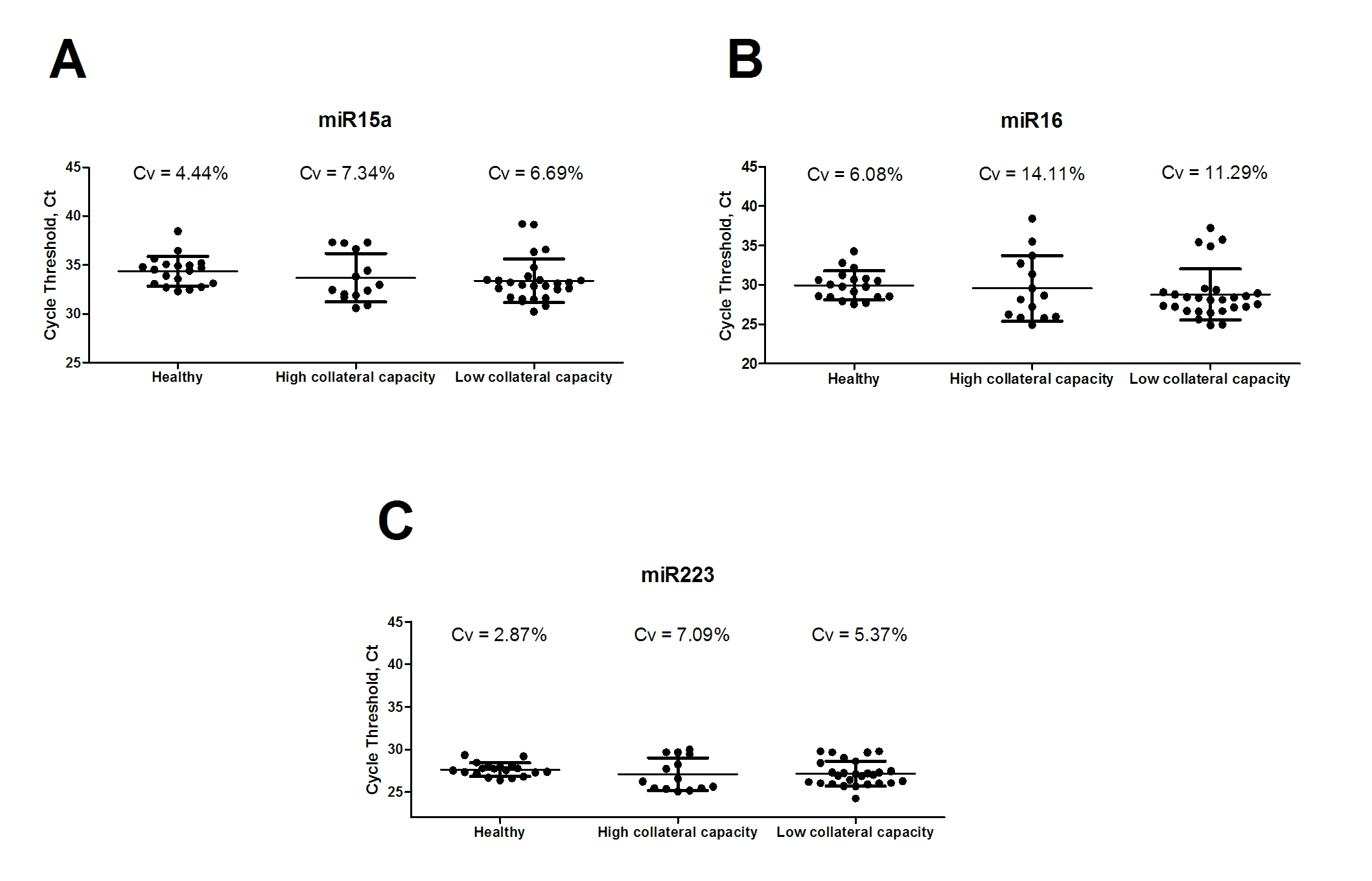

Supplement: S1 Fig — Cycle threshold values of three reference miRNA candidates (A: miR15a; B: miR16; C: miR223) in patients with low (CFI < 0.39) and high (CFI > 0.39) collateral capacity, as well as healthy individuals. MiR223 demonstrates most stable expression between the two patient groups and healthy controls, with the lowest coefficient of variation (Cv) values. Data are presented as mean ± SD. CFI: collateral flow index; miRNA: microRNA (TIF) [file pone.0137035.s001.tif]

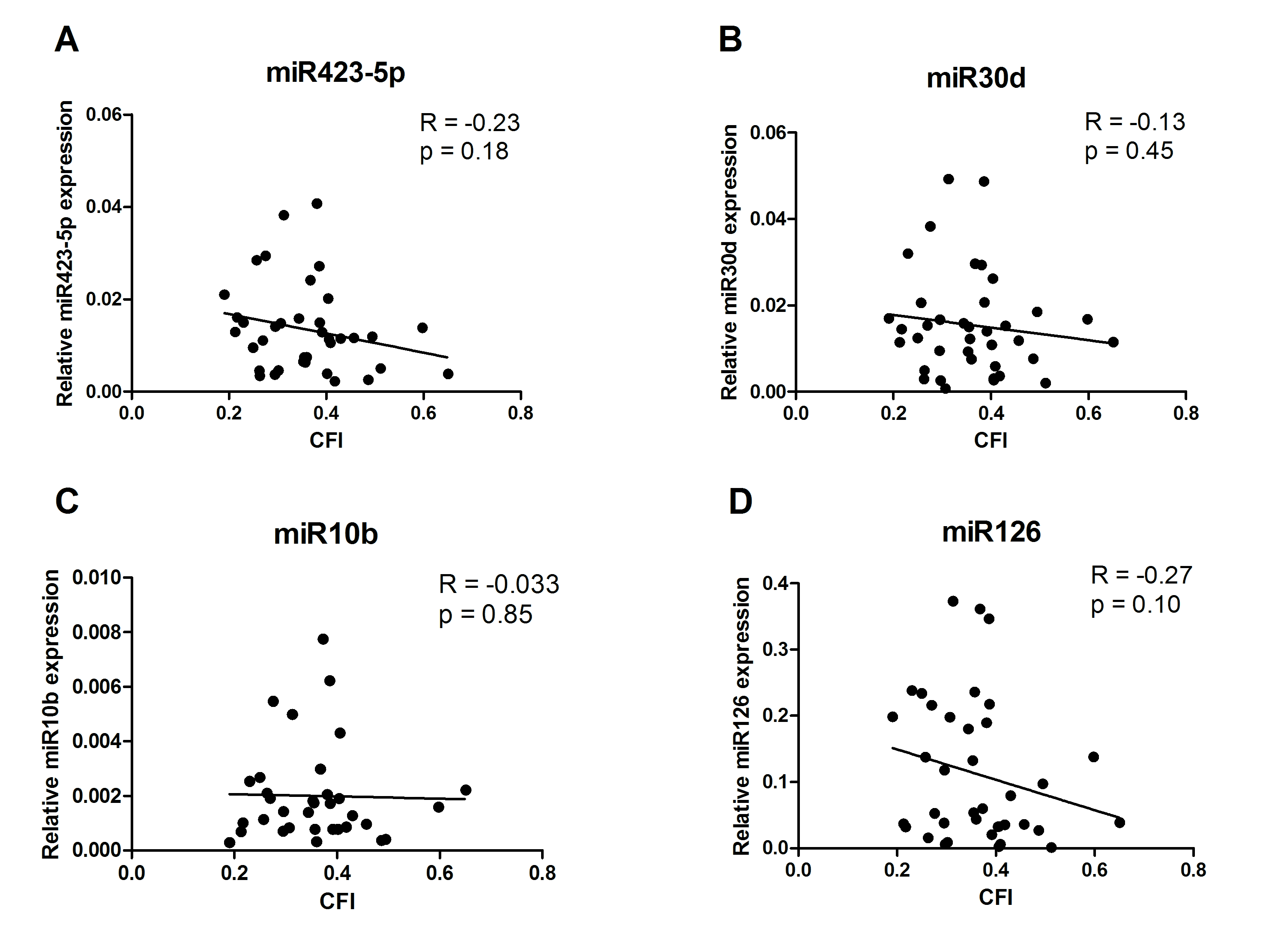

Supplement: S2 Fig — No significant correlation seen between relative expression levels of selected microRNAs (A: miR423-5p, B: miR30d, C: miR10b, D: miR126) and collateral flow index (CFI) values. (TIF) [file pone.0137035.s002.tif]
