# Supplementary material for: Circulating MicroRNAs Characterizing Patients with Insufficient Coronary Collateral Artery Function
Source: PLoS One. 2015 Sep 2;10(9):e0137035. doi: 10.1371/journal.pone.0137035 (PMC4558025; doi:10.1371/journal.pone.0137035)
Supplement: S1 Table — Correlation of circulating miRNAs with circulating leukocyte groups. Significant correlation was noted between miR10b and lymphocyte levels as well as between miR10b and monocytes. (DOCX) [file pone.0137035.s003.docx]

**S2 Table Correlation between leukocytes and respective miRNAs in all CTO patients (n=41).**

|  |  |  |
| --- | --- | --- |
|  | **R** | **p-value** |
| **miR423-5p** |  |  |
| WBC (10^9^/L) | 0.07 | 0.67 |
| Thrombocytes (10^9^/L) | -0.08 | 0.63 |
| Neutrophils (10^9^/L) | 0.008 | 0.96 |
| Eosinophils (10^9^/L) | 0.06 | 0.72 |
| Basophils (10^9^/L) | 0.09 | 0.61 |
| Lymphocytes (10^9^/L) | 0.08 | 0.62 |
| Monocytes (10^6^/L) | 0.21 | 0.22 |
| **miR30d** |  |  |
| WBC (10^9^/L) | 0.11 | 0.51 |
| Thrombocytes (10^9^/L) | -0.12 | 0.47 |
| Neutrophils (10^9^/L) | 0.03 | 0.85 |
| Eosinophils (10^9^/L) | -0.14 | 0.41 |
| Basophils (10^9^/L) | 0.08 | 0.64 |
| Lymphocytes (10^9^/L) | 0.17 | 0.32 |
| Monocytes (10^6^/L) | 0.17 | 0.32 |
| **miR10b** |  |  |
| WBC (10^9^/L) | 0.32 | 0.07 |
| Thrombocytes (10^9^/L) | 0.04 | 0.81 |
| Neutrophils (10^9^/L) | 0.12 | 0.51 |
| Eosinophils (10^9^/L) | 0.003 | 0.99 |
| Basophils (10^9^/L) | -0.19 | 0.28 |
| Lymphocytes (10^9^/L) | 0.38 | 0.03* |
| Monocytes (10^6^/L) | 0.37 | 0.04* |
| **miR126** |  |  |
| WBC (10^9^/L) | 0.06 | 0.71 |
| Thrombocytes (10^9^/L) | -0.26 | 0.12 |
| Neutrophils (10^9^/L) | 0.06 | 0.73 |
| Eosinophils (10^9^/L) | -0.06 | 0.73 |
| Basophils (10^9^/L) | 0.05 | 0.79 |
| Lymphocytes (10^9^/L) | 0.12 | 0.49 |
| Monocytes (10^6^/L) | 0.26 | 0.12 |

Correlation of circulating miRNAs with circulating leukocyte groups. Significant correlation was noted between miR10b and lymphocyte levels as well as between miR10b and monocytes.
